# Fluorescence *In situ* Hybridization: Cell-Based Genetic Diagnostic and Research Applications

**DOI:** 10.3389/fcell.2016.00089

**Published:** 2016-09-05

**Authors:** Chenghua Cui, Wei Shu, Peining Li

**Affiliations:** ^1^Laboratory of Clinical Cytogenetics, Department of Genetics, Yale School of Medicine New Haven, CT, USA; ^2^Department of Pathology, Institute of Hematology and Blood Diseases Hospital, Chinese Academy of Medical Sciences Tianjin, China; ^3^Department of Cell Biology and Genetics, Guangxi Medical University Nanning, China

**Keywords:** fluorescence *in situ* hybridization (FISH), genetic diagnosis, aneuploidy, pathogenic copy number variants (CNV), microdeletion/microduplication syndromes, Cas-9 mediated FISH (CASFISH), oligopaint-FISH, single molecule RNA FISH (smRNA-FISH)

## Abstract

Fluorescence *in situ* hybridization (FISH) is a macromolecule recognition technology based on the complementary nature of DNA or DNA/RNA double strands. Selected DNA strands incorporated with fluorophore-coupled nucleotides can be used as probes to hybridize onto the complementary sequences in tested cells and tissues and then visualized through a fluorescence microscope or an imaging system. This technology was initially developed as a physical mapping tool to delineate genes within chromosomes. Its high analytical resolution to a single gene level and high sensitivity and specificity enabled an immediate application for genetic diagnosis of constitutional common aneuploidies, microdeletion/microduplication syndromes, and subtelomeric rearrangements. FISH tests using panels of gene-specific probes for somatic recurrent losses, gains, and translocations have been routinely applied for hematologic and solid tumors and are one of the fastest-growing areas in cancer diagnosis. FISH has also been used to detect infectious microbias and parasites like malaria in human blood cells. Recent advances in FISH technology involve various methods for improving probe labeling efficiency and the use of super resolution imaging systems for direct visualization of intra-nuclear chromosomal organization and profiling of RNA transcription in single cells. Cas9-mediated FISH (CASFISH) allowed *in situ* labeling of repetitive sequences and single-copy sequences without the disruption of nuclear genomic organization in fixed or living cells. Using oligopaint-FISH and super-resolution imaging enabled *in situ* visualization of chromosome haplotypes from differentially specified single-nucleotide polymorphism loci. Single molecule RNA FISH (smRNA-FISH) using combinatorial labeling or sequential barcoding by multiple round of hybridization were applied to measure mRNA expression of multiple genes within single cells. Research applications of these single molecule single cells DNA and RNA FISH techniques have visualized intra-nuclear genomic structure and sub-cellular transcriptional dynamics of many genes and revealed their functions in various biological processes.

## Introduction

Fluorescence *in situ* hybridization (FISH) uses DNA fragments incorporated with fluorophore-coupled nucleotides as probes to examine the presence or absence of complementary sequences in fixed cells or tissues under a fluorescent microscope. This hybridization-based macromolecule recognition tool was very effective in mapping genes and polymorphic loci onto metaphase chromosomes for constructing a physical map of the human genome (Langer-Safer et al., 1982; Lichter et al., 1993). FISH technology offers three major advantages including high sensitivity and specificity in recognizing targeted DNA or RNA sequences, direct application to both metaphase chromosomes and interphase nuclei, and visualization of hybridization signals at the single-cell level. These advantages increased the analytic resolution from Giemsa bands to the gene level and enabled rapid detection of numerical and structural chromosomal abnormalities (Klinger et al., 1992; Ried et al., 1992). Clinical application of FISH technology had upgraded classical cytogenetics to molecular cytogenetics. With the improvement in probe labeling efficiency and the introduction of a super resolution imaging system, FISH has been renovated for research analysis of nuclear structures and gene functions. This review presents the recent progress in FISH technology and summarizes its diagnostic and research applications.

## Cell based genetic diagnosis by FISH

### Analytical and clinical validities and practice guidelines

**Figure 1:**
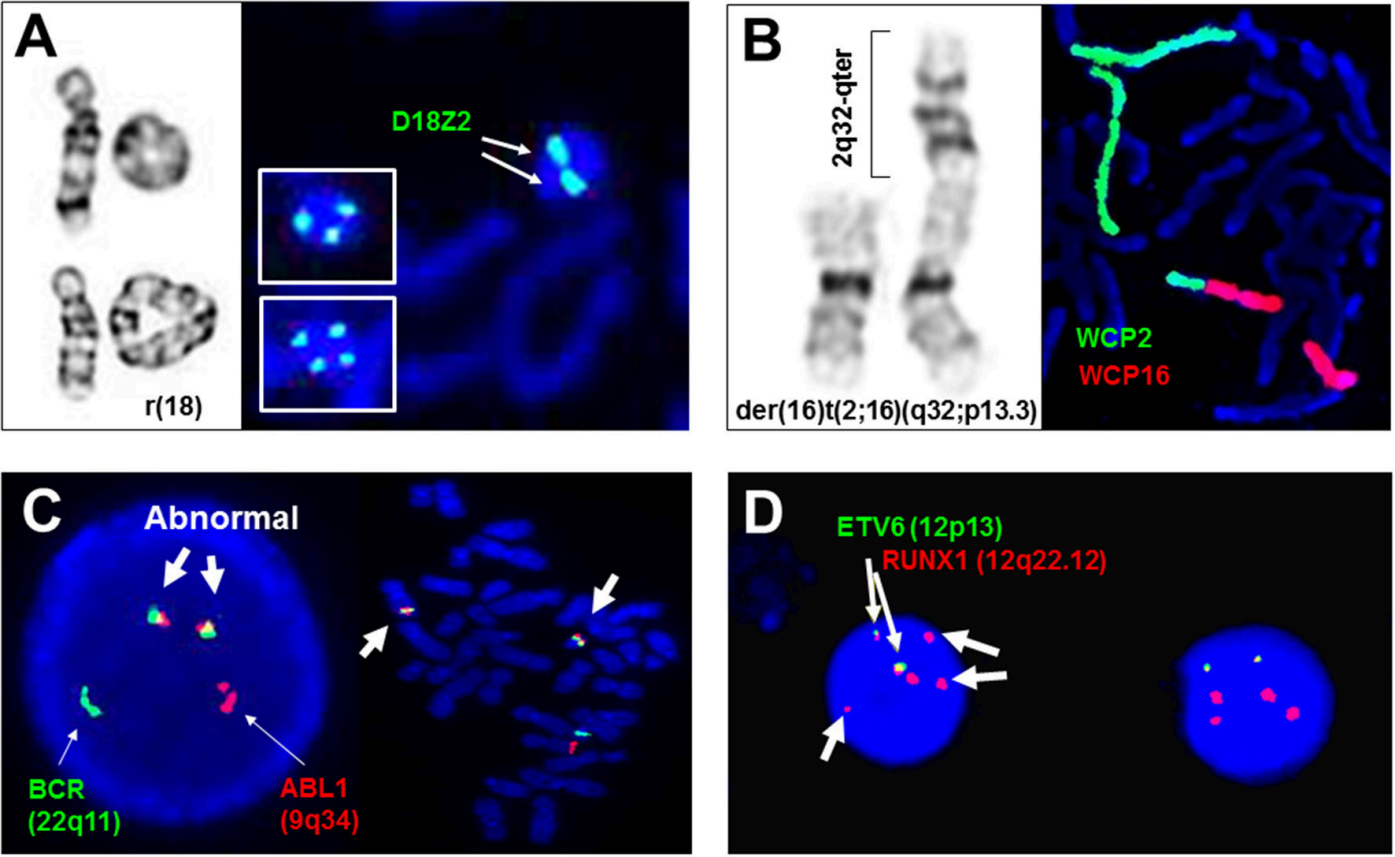
**Adjunctive and diagnostic assays of FISH in clinical cytogenetics. (A)** The detection of di-centric, tri-centric, and tetra-centric ring chromosome 18 using a centromeric probe D18Z2 for chromosome 18. Left panel shows normal chromosome 18, dicentric ring 18 in top, and tetracentric ring 18 in bottom, right panel shows dicentric ring 18 and tricentric/tetracentric ring 18 in insets by FISH. **(B)** The detection of a derivative chromosome 16 from a 2q32/16p13.3 translocation by whole chromosome painting probes for chromosomes 2 (WCP2) and 16 (WCP16). **(C)** The detection of ABL1/BCR gene fusions in interphase and metaphase cells by dual color double fusion probes (thin arrows point to the normal signal and thick arrows point to the abnormal fusion signals). **(D)** Diagnostic use of ETV6 and RUNX1 probes for the detection of two fusion signals for a cryptic t(12;21)(p13;q22), loss of an ETV6 signal and gain of three extra RUNX1 signals (thin arrows point to the fusion signals and thick arrows to extra RUNX1 signals). All images are from Yale clinical cytogenetics laboratory.

Most DNA fragments used as probes are extracted from bacterial artificial clones (BACs) which contain cloned human genomic DNA sequences in the size of 100–200 Kilobases (Kb). These DNA fragments could be directly labeled by nick translation to incorporate nucleotides coupled with different fluorophores such as coumarins, fluoresceins, rhodamine, and cyanines (Cy3, Cy5, and Cy7) (Morrison et al., 2003). According to the targeted regions and labeling design, FISH probes can be divided into locus-specific probes targeted to specific regions or genes and regional painting probes for specific chromosomal bands, an entire chromosome or whole genome. Commonly used locus-specific probes include alpha repetitive sequences for centromeric regions and single copy sequences for subtelomeric and gene regions. Multi-color locus-specific probes allow simultaneously detection of numerical abnormalities of two to three regions in one FISH assay. For structural rearrangements, locus-specific probes with different fluorophores for two genes or for the 5′ and 3′ regions of a gene have been used to detect “double-fusion” signals resulting from a reciprocal translocation or “break apart” signals from a gene rearrangement, respectively. Painting probes have been used mostly in a research setting to dissect chromosome domains within a nucleus or structural rearrangements in metaphase chromosomes. Figure 1 shows representative FISH applications of locus-specific and chromosome painting probes in the detection of numerical and structural chromosomal abnormalities.

Earlier studies had evaluated signal-to-noise ratios, spatial resolution of the fluorescent signals, and hybridization/detection efficiencies of FISH tests on lymphocytes and aminocytes (Klinger et al., 1992; Ried et al., 1992). These studies led to the commercialization of FISH probes with optimized probe selection and standardized labeling, and the clinical utility of FISH testing in large case series (Ward et al., 1993). To ensure safe and effective diagnostic application, a clinical cytogenetics laboratory needs to establish the analytical and clinical validities for every FISH assay. The analytical validity of a FISH assay is evaluated by its targeted accuracy, sensitivity, specificity, and normal reference ranges following a standardized laboratory procedure (Wolff et al., 2007; Ciolino et al., 2009). FISH testing could be used as an adjunctive assay or a stand-alone diagnostic assay for constitutional and somatic abnormalities. The clinical validity for its intended use should be evaluated by calculating the sensitivity from patients with targeted abnormalities and the specificity from normal controls. Other analytical and clinical considerations include possible false positive or negative results, continuous monitoring of signal variations, periodical evaluation and batch-to-batch comparisons of probe performances (Test and Technology Transfer Committee, 2000).

FISH technology enabled the detection of an increased spectrum of genetic disorders from chromosomal abnormalities to submicroscopic copy number variants (CNVs) and extended the cell-based analysis from metaphases to interphases (Xu and Li, 2013). The analytical resolution of FISH is in the range of 100–200 Kb as determined by the probe size, which is 50-fold higher than the 5–10 megabase (Mb) Giesma banding of a high resolution karyotyping. Locus-specific probes detected submicroscopic CNV and led to the identification of a group of genomic disorders (also termed contiguous gene syndromes or microdeletion syndromes), such as DiGeorge syndrome (OMIM#188400) by a deletion at 22q11.2, Prader-Willi syndrome (OMIM#176270) and Angelman syndrome (OMIM#105830) by a deletion at 15q11.2. FISH can be performed directly on interphase nuclei, which eliminated the time consuming cell culture procedure and extended its diagnostic application toward rapid screening of chromosomal and genomic abnormalities. In the following sections, the diagnostic applications of FISH technology are focused on three main areas: prenatal screening and postnatal diagnosis of constitutional chromosomal abnormalities and submicroscopic pathogenic CNVs, identification and monitoring of acquired chromosomal abnormalities in hematopoietic and solid tumors, and the detection of infectious diseases caused by microbials and parasites.

### Detection of constitutional chromosomal abnormalities and pathogenic CNVs

A Multiplex FISH panel with differentially labeled probes has been developed for prenatal screening of common aneuploidies involving gains or losses of chromosomes X, Y, 13, 18, and 21 (Ried et al., 1992; Ward et al., 1993). Pregnant women with a single indication or combined indications of advanced maternal age, abnormal ultrasound findings, or abnormal maternal serum screening have an increased risk of 4–30% for carrying numerical and structural chromosomal abnormalities; among these abnormalities, 84% were numerical abnormalities mostly detectable by the multiplex FISH panel, and 16% were structural abnormalities required further microarray analysis (Li et al., 2011). For prenatal cases with cardiac anomalies detected by prenatal ultrasound examination, DiGeorge syndrome was detected by FISH. Recently, the application of non-invasive prenatal testing by massive parallel sequencing on maternal cell-free fetal DNA significantly improved the accuracy of aneuploidy screening, which resulted in a 57% decline in invasive prenatal procedures and an increase of diagnostic yield of chromosomal abnormalities (Xu Z. Y. et al., 2013; Meng et al., 2015). Despite these technology advances in prenatal diagnosis, the multiplex FISH panel is still used as an adjunctive assay for rapid detection of common aneuploidies. It should be noted that false positive or negative results as well as maternal cell contamination have been noted in prenatal FISH analysis. Therefore, an irreversible therapeutic action should not be initiated on the basis of FISH results alone. The current guideline recommended that clinical decisions should be made based on two of three pieces of available information: FISH results, conventional cytogenetic analysis and clinical information (Test and Technology Transfer Committee, 2000). Furthermore, aneuploidies and polyploidies have been detected in about 50% of first trimester spontaneous abortions by chromosome analysis and in 35% of products of conception culture failure cases by microarray analysis; it is recognized that an extended FISH panel for chromosomes X/Y/18, 13/21, and 15/16/22 will detect all polyploidies, 84% of aneuploidies, and 69% of multiple aneuploidies causing miscarriages (Zhou et al., 2016).

Developmental delay, intellectual disabilities, and multiple congenital anomalies are present in 1–5% of newborns, and chromosome microarray analysis as the first tier genetic testing has detected a spectrum of cytogenomic abnormalities in 10~20% of these patients (Miller et al., 2010; Li et al., 2015). Analysis of abnormal findings from consecutive pediatric cases observed genomic disorders (microdeletion/microduplication syndromes), subtelomeric rearrangements, interstitial imbalances, chromosomal structural rearrangements, and aneuploidies in about 37, 26, 19, 10, and 8% of these cases, respectively (Xu et al., 2014). Cell-based FISH testing has been a cost-effective adjunctive assay to confirm microarray detected genomic disorders and then to detect carrier statues in a follow-up parental study. Microdeletions can be detected as a loss of one signal in metaphases and interphases, while microduplications can be detected as “twin-spot” like two signals in the interphase nuclei. For subtelomeric rearrangements, a complete set of subtelomeric FISH probes for all human chromosomes was developed (Ning et al., 1996) and have been used routinely as an adjunctive assay in visualizing cryptic and complex subtelomeric rearrangements (Li et al., 2006; Rossi et al., 2009). For many newly defined loci of genomic disorders and interstitial imbalances, there are no commercially available FISH probes. Therefore, “home-brew” targeted BAC clone FISH probes were used for these unique cases (Li et al., 2006; Khattab et al., 2011).

Structural rearrangements like ring chromosomes and small supernumerary marker chromosomes (sSMC) present not only segmental gains or losses but also a mosaic pattern due to their dynamic behavior in mitosis. As shown in Figure 1A, centromeric FISH probes are routinely used to track the changes from dicentric, tricentric, and tetracentric ring chromosomes to loss of the ring through mitosis. Subtelomeric and interstitial FISH probes have been used to define the intactness of the ring chromosome and the level of mosaicism (Zhang et al., 2004; Xu F. et al., 2013). A cytogenomic approach combining chromosome, FISH, and microarray analyses has been recommended for characterizing the genomic structure, mitotic instability, and mechanisms of ring formation for cases with a ring chromosome (Zhang et al., 2012). sSMC are extra centric chromosome fragments usually in the forms of an inverted duplication or a small ring chromosome and present in 0.043% of newborn children. Several sSMC have syndromic phenotypes such as inv dup(22q11.2) for cat-eye syndrome (OMIM*607576) and i(12p) for Pallister Killian syndrome (OMIM#601803), and others like inv dup(15q) and i(18p) can have variable phenotypes (Liehr et al., 2004, 2006). About 30% of sSMC are derived from chromosome 15; the D15S10 or *SNRPN* probes are routinely used to assess inv dup(15q) (Wang et al., 2015). The euchromatic material in sSMC can be detected by a microarray analysis. A set of pericentric core probes for each arm of human chromosomes has been validated for characterizing unambiguously the chromosomal origin of sSMC and the level of mosaicism (Castronovo et al., 2013).

### Identification and monitoring of acquired chromosomal abnormalities

The discovery of Philadelphia chromosome in chronic myeloid leukemia (CML) followed by the characterization of t(9;22)(q34;q11) with underlying ABL1/BCR gene fusions supported the causative role of chromosomal abnormalities in carcinogenesis and set the foundation for cancer cytogenetics (Mitelman et al., 2007). Cancer is considered a genetic disease at the cellular level resulting from either a progressive process or a one-off catastrophic event (Stephens et al., 2011; Li and Cui, 2016). The two main pathogenetic pathways for hallmarks of cancer development are the inactivation of tumor suppressor genes by deletions, mutations, miRNA upregulation, or epigenetic mechanisms, and the activation or deregulation of oncogenes as a consequence of point mutations, amplification or balanced cytogenetic abnormalities (Vogelstein and Kinzler, 2004; Hanahan and Weinberg, 2011). Recurrent chromosomal abnormalities including translocations, deletions, duplications, and gene amplifications associated with distinct tumor entities have been characterized; specifically designed FISH panels have been widely used in the diagnosis and monitoring of acquired chromosomal abnormalities in hematologic and solid tumors (Hu et al., 2014; Liehr et al., 2015; Mikhail et al., 2016).

Current guidelines recommend an integrated approach for cancer cytogenetic diagnosis (Wolff et al., 2007). In general, both conventional karyotyping and FISH testing are used for initial diagnosis and follow up monitoring of clonal abnormalities. For hematopoietic and lymphoid tumors, the most commonly used FISH probes and disease-specific panels in a clinical cytogenetics laboratory are listed in Table 1. Results from a FISH panel offer a quick evaluation of targeted abnormal patterns and their percentage within the bone marrow cells or leukocytes. Chromosome analysis will then reveal the clonal abnormalities and clonal evolution. For leukemias requiring urgent treatment, such as acute promyelocytic leukemia (APL) caused by the t(15;17)(q24;q21) with underlying PML/RARa fusions, rapid FISH result is mandated for the administration of all-trans retinoic acid (ATRA). Targeted therapy against the ABL1/BCR fusion protein by small molecule tyrosine inhibitors like imatinib mesylate (Gleevec), dasatinib (Sprycel), and nilotinib (Tasigna) has increased the 10-year overall survival from 20 to 80–90% (Li et al., 2013). For many cryptic rearrangements undetectable by routine chromosome analysis, such as t(12;21)(p13;q22) with ETV6/RUNX1 gene fusions, t(4;14)(p16.3;q32) with FGFR3/IGH gene fusions, deletions of 12p13 (ETV6), 13q14 (RB1), and 17p13 (TP53), FISH tests are considered a stand-alone diagnostic assay. Adjunctive use of FISH probes to further define ambiguous or hidden chromosomal abnormalities is required for many cases (Kamath et al., 2008; Massaro et al., 2011). Additionally, FISH is a sensitive and timely method to monitor residual diseases with known clonal abnormality and bone marrow transplantation by sex-mismatch donor at cellular level. Considering some hematologic tumors may be morphologically similar and the abnormalities may not be detected by low-resolution karyotyping and/or in low percentage of leukemic cells, FISH could be important for differential diagnosis between these diseases. For example, cyclin D1 (*CCND1*) translocation can be detected by FISH as a characteristic abnormality in mantle cell lymphoma, which provides differential diagnosis for morphologically similar chronic lymphoid leukemia (CLL). Furthermore, FISH for nuclear DNA can be combined with immunostaining of cytoplasmic markers for simultaneous identification of chromosomal abnormalities and cell types. For example, *IGH* translocation is present in multiple myeloma and monoclonal gammopathy of undetermined significance (MM/MGUS) with high frequency, which is usually detected in plasma cells. In a two-step assay with first the hybridization of *IGH* probe and then immune-staining by fluoresceinisothiocyanate (FITC)-conjugated antibodies against κ- or λ-light chain, the FITC-stained cytoplasm and *IGH* break apart signals within the nuclei were visualized in plasma cells simultaneously. This modified immuno-FISH was expected to improve the diagnostic accuracy but the low sensitivity limited its application only in follow-up study (Boersma-Vreugdenhil et al., 2003).

**Table 1:** **List of FISH panels and probes for hematopoietic and lymphoid tumors**.

**Gene (G-band)**	**Probe Design**	**Myeloid leukemia**			**Lymphocytic leukemia**			**Lymphoma**	**MM/MGUS**	**MPD**
		**CML**	**MDS**	**AML**	**CLL**	**B-ALL**	**T-ALL**			
CKS1B (1q21), CDKN2C (1p32)	DCE								1p/1q+	
PBX1 (1q23.3), TCF3 (19p13.3)	DCDF					t(1;19)		t(1;19)		
ALK (2p23)	DCBAP							ALK		
MECOM (3q26)	DCBAP			inv(3)						
BCL6 (3q27)	DCBAP							BCL6		
D4Z1 (4cen), D10Z1 (10cen), D17Z1 (17cen)	TCE					+4/10/17				
PDGFRA (4q12)	DCBAP									PDGFRA
FGFR3 (4p16.3), IGH (14q32)	DCDF								t(4;14)	
TAS2R1 (5p15.31), EGR1 (5q31)	DCE		5q−/−5							
PDGFRB (5q33)	DCBAP					PDGFRB				PDGFRB
MYB (6q23), D6Z1 (6cen)	DCE								6q−	
RELN (7q22), TES (7q31)	DCE		7q−/−7							
TCRB (7q34)	DCBAP						TCRB			
FGFR1 (8p11)	DCBAP									FGFR1
RUNX1T1 (8q21), RUNX1 (21q22)	DCDF			t(8;21)						
cMYC (8q24)	DCBAP							cMYC		
cMYC (8q24), D20S108 (20q12)	DCE		+8/20q−							
PAX5 (9p13.2)	DCBAP					PAX5				
CDKN2A (9p21), D9Z3 (9cen)	DCE					9p−	9p−			
ABL (9q34), BCR (22q11)	DCDF	t(9;22)				t(9;22)	t(9;22)			
CCND1 (11q13), IGH (14q32)	DCDF							t(11;14)	t(11;14)	
ATM (11q22), TP53 (17p13)	DCE				11q−/17p−					
KMT2A (11q23)	DCBAP			KMT2A		KMT2A	KMT2A			
ETV6 (12p13), RUNX1 (21q22)	DCDF					t(12;21)				
DLEU1 (13q14), D13S25 (13q34)	DCE								13q−	
DLEU1 (13q14), D13S25 (13q34), D12Z3 (12cen)	TCE				13q−/+12					
TCRA/D (14q11)	DCE						TCRA			
IGH (14q32)	DCBAP				IGH			IGH	IGH	
IGH (14q32), BCL2 (18q21)	DCDF							t(14;18)		
SNRPN (15q11.2), TP53 (17p13)	DCE								+15/17p−	
PML (15q24), RARA (17q21)	DCDF			t(15;17)						
MYH11 (16p13), CBFB (16q22)	DCDF			inv(16)						
MALT1 (18q21)	DCBAP							MALT1		
CRLF2 (Xp22.33)	DCBAP					CLFR2				

*DCE,dual-color enumerate; TCE, tri-color enumerate; DCBAP, dual-color break apart; DCDF, dual-color double fusion; CML, Chronic myeloid leukemia; MDS, Myelodysplastic syndrome; AML, Acute myeloid leukemia; CLL, Chronic lymphocytic leukemia; B-ALL, B-cell acute lymphocytic leukemia; T-ALL, T-cell acute lymphocytic leukemia; MM/MGUS, Multiple myeloma/Monoclonal mopathy of undetermined significance; MPD, Myeloproliferative disorder. Shaded for recurrent abnormalities detected by a primary FISH panel, unshaded for secondary FISH probes for specific abnormalities. For references see (Hu et al., 2014; Liehr et al., 2015), and (Mikhail et al., 2016)*.

FISH tests are widely used in various types of solid tumors. For example, FISH can define gene rearrangements in congenital fibrosarcoma with a novel complex translocation (Marino-Enriquez et al., 2008) and validate subclone markers in heterogeneous melanoma biopsies (Parisi et al., 2011). FISH results can be used to guide cancer treatment. For example, Herceptin-targeted therapy is effectively against *HER2* over-expressed breast cancer. For routine clinical specimen, immunohistochemistry, real-time polymerase chain reaction, and FISH were used to assess the *HER2* protein level, RNA expression, and DNA copy numbers, respectively. Among these methods, FISH offered a cell-based evaluation for the ratio of *HER2* gene copy number to the number of copies of chromosome 17 (*HER2*/CEP17 ratio). The FISH scoring criteria for *HER2*/CEP17 ratio and the interpretive guidelines were reported (Hicks et al., 2005). Many targeted therapies for recurrent translocations in various types of solid tumors have been either approved by FDA or are under clinical trials. For example, lapatinib, sorafenib, sunitinib, termsirolimus, and pazopanib have been used for papillary renal cell carcinoma with translocations involving the *TFE3* gene at Xp11.2; cixutumumab and mithramycin are in phase II clinical trial for Ewing sarcoma with translocation involving the EWSR1 gene at 22q12 (Li et al., 2013). FISH assays using probes for specific recurrent translocations from different solid tumors could guide effective targeted therapy. FISH tests were also used to evaluate sperm aneuploidy frequencies before and after chemotherapy in patients with testicular cancer and Hodgkin's lymphoma; significantly increased frequencies of aneuploidies for a duration up to 24 months were noted (De Mas et al., 2001; Tempest et al., 2008). It was recommended that genetic counseling about potentially increased reproduction risk from chemotherapy should be offered to cancer patients.

### Detection of infectious diseases by FISH

The majority of FISH probes target to specific chromosomal and genomic abnormalities in the human genome. Rapid phylogenetic identification of single microbial cells was achieved using fluorescently labeled oligonucleotides complementary to 16S ribosomal RNA (rRNA) (DeLong et al., 1989). Some segments in the 16S rRNA are invariant in all organisms but phylogenetic group-specific 16S rRNA in different groups of organism can be used as oligonucleotide FISH probes (length 17–34 nucleotides) to identify infectious agents in clinical samples. For example, FISH probes complementary to specific sequence of 16s rRNA can detect malaria infection in blood samples. The *Plasmodium* Genus (P-Genus) FISH assay has a *Plasmodium* genus specific probes that detect all five species of *Plasmodium* known to cause the disease in humans. The sensitivity of this FISH assay is better than the Giemsa staining method. A LED light source may be an available device to read FISH result, which can extend the clinical application of FISH especially in the resource-limited areas. Since rRNA has a short life and is present in a live organism with plenty of copies, FISH should be done in the live pathogens (Shah et al., 2015).

## Single-cell DNA structural and RNA transcriptional analyses

FISH assays using locus-specific and regional painting probes are still a powerful tool in visualizing simple and complex chromosomal and genomic rearrangements. Fiber-FISH by locus-specific BAC clone probes within a 900 Kb 17q12 inversion hybridizing onto stretched DNA fibers correlated the inversion orientations with associated haplotypes, which allowed the evaluation of inversion frequencies among human populations globally (Donnelly et al., 2010). Pericentriomeric heterochromatin probes were used in a three dimensional FISH (3D-FISH) to study intra-nuclear centromeric positions in cultured cells from patients with ICF syndrome (immunodeficiency, centromeric region instability, facial anomalies) and Robert syndrome (cohesion defect by mutations in the *ESCO2* gene) (Dupont et al., 2012, 2014). Multi-color FISH (M-FISH) by painting probes specific for a human chromosome and multi-color banding FISH (M-BAND) by painting probes specific for every band in a chromosome were used to visualize complex chromosomal rearrangements from chromothripsis in two patients with acute myeloid leukemia (Mackinnon and Campbell, 2013). Chromothripsis are seen as regional clustering of breakpoints and regularity of oscillating copy-number states by microarray analysis and as heterogeneous staining regions, marker or ring chromosomes, and other undefinable rearrangements by chromosome analysis (Stephens et al., 2011). Selected FISH probes targeting to the oscillating copy-number gains and losses could be used to monitor the abnormal clones with chromothripsis.

**Figure 2:**
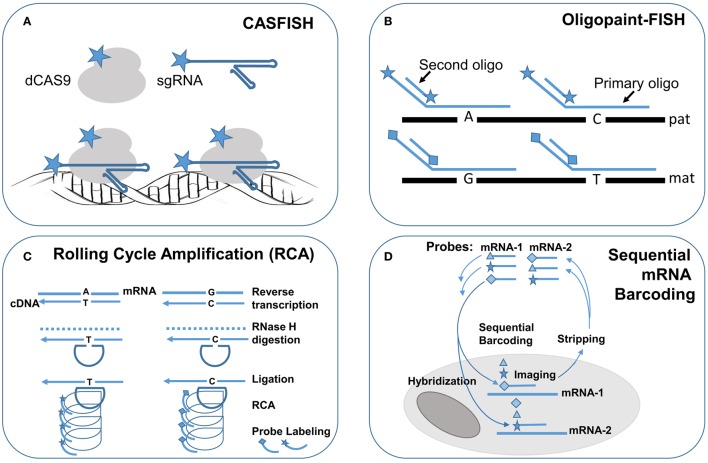
**Single molecule FISH techniques for research application in single cells**. Schematic drawings of single molecule FISH methods: **(A)** Cas-9 mediated FISH (CASFISH) using fluorophore-coupled sgRNAs and dCAS9. **(B)** Oligopaint-FISH using fluorophore-coupled primary oligonucleotides for targeted SNP loci and fluorophore-coupled second oligonucleotide to enhance labeling efficiency shows differential labeling of paternal (pat) and maternal (mat) chromosomes. **(C)** single molecule RNA FISH by rolling cycling amplification (RCA) using padlock probes targeting to reverse transcripted cDNA with different alleles followed by ligation, cycling amplification and specific fluorophore-couple probe hybridization and visualization. **(D)** Sequential barcoding of multiplex different mRNAs by repeat rounds of hybridization, imaging, and stripping. Star, diamond, and triangle are symbols for different fluorophores.

FISH technology has made significant progress with the innovation of novel labeling methods and the introduction of super resolution imaging systems for fine mapping of intra-nuclear genomic structures and for single cells single molecule profiling of cytoplasmic RNA transcription. Recently, a novel FISH method using nuclease-deficient clustered regularly interspaced short palindromic repeats (CRISPR)/CRISPR-associated caspase 9 (dCas9) system was developed. The initial design used enhanced green fluorescent protein (EGFP) tagged dCas9 and small guide RNA (sgRNA) targeting to repetitive telomere sequences or sgRNAs tiling along a non-repetitive genomic sequences at the *MUC4* locus. This method enabled the visualization of intra-nuclear locations and dynamics of telomeres and *MUC4* loci during mitosis in living human cells (Chen et al., 2013). Further modification by using both fluorophore-coupled sgRNA and fluorophore-coupled dCas9 was termed Cas9-mediated FISH (CASFISH); rapid and robust labeling of repetitive DNA elements in preicentromere, centromere, G-rich telomere, and *MUC4* gene by CASFISH was demonstrated (Figure 2A; Deng et al., 2015). This CASFISH did not require the denature treatment for targeted DNA and therefore preserved the nature spatiotemporal organization of the nucleus. The CASFISH process is remarkably rapid (within 1 h) and can be used directly on fixed tissues or living cells. However, using tiling sgRNAs for single-copy gene regions could have low labeling efficiency and higher background. Further optimization of this CASFISH technology is needed before its application for basic research and genetic diagnosis.

A synthesized primary single-strand oligonucleotide library targeting to a single copy region of the genome along with fluorophore-coupled second oligonucleotides complementary to a portion of the primary oligonucleotides were developed for so-called oligopaint FISH (Beliveau et al., 2015). Co-hybridization of a set of hundreds to thousands of primary fluorophore-coupled oligopaint probes (30–42 bases in length for targeted genome region and hinged 14–32 bases for second oligonucleotides) with fluorophore-coupled second oligonucleotide (14–32 bases) can visualize a 52 Kb–3 Mb regions in nuclei with a 96–100% hybridization efficiency. Oligopaint FISH probes designed with one fluorophore for specified single nucleotide polymorphisms (SNPs) in a targeted region from one chromosome and another fluorophore for these SNPs in the homology chromosome enabled differential labeling of the two homologous chromosomes. Stochastic optical reconstruction microscope (STORM) was used for single-molecule super-resolution imaging. Therefore, with prior information of the specific SNP alleles from the two homologous chromosomes, oligopaint FISH showed *in situ* haplotyping for paternal and maternal chromosomes (Figure 2B). The oligopaint probes are chosen bioinformatically to avoid repetitive DNA sequences and they can be selected to target any organisms whose genomes have been sequenced. With further improvement on signal pattern recognition from the SNP loci, oligopaint FISH should enable direct analysis of fine-scale chromatin structure, differential visualization of homologous chromosomes, and allele-specific studies of gene expression.

RNA FISH is a cell-based technique for detecting mRNA transcripts. With the advance of various methods for signal amplification and super-resolution imaging, single molecule RNA FISH (smRNA-FISH) techniques have been developed. Several approaches, including branched DNA probes, tyramide signal amplification, quantum dots, and padlock-rolling circle amplification (RCA), have been used for signal enhancement (Kwon, 2013). RCA is the only method capable of distinguishing single nucleotide allelic changes in transcripts. Briefly, reverse transcription was performed *in situ* on cells and tissue sections to generate complementary DNA (cDNA), the mRNA was degraded by ribonuclease H, and then padlock probes were hybridized to targeted cDNA with 5′ and 3′ arms circularized by a T4 DNA ligase. The circularized padlock probes served as a template for RCA by Φ29 DNA polymerase, and then fluorophore-couple oligonucleotide probes specific for each padlock probe could be hybridized and visualized (Figure 2C; Larsson et al., 2010). To increase the capacity for multiplex detection of different mRNA molecules in single cells, combinatorial labeling, and optical super-resolution microscope were used to measure mRNA levels of 32 genes simultaneously in single *Saccharomyces cerevisiae* cells (Lubeck and Cai, 2012). Further modification introduced a sequential barcoding scheme for multiplex different mRNA quantitation (Lubeck et al., 2014). In this scheme, the mRNAs in cells were barcoded by sequential rounds of hybridization, imaging and probe stripping (Figure 2D). Theoretically, the multiplexing capacity scaled up quickly as the number of fluorophores and rounds of hybridization increased. In practice, the available fluorophores were limited and each round of hybridization introduced loss of the RNA integrity in the tested cells.

Various smRNA-FISH methods have been used in imaging cell-type specific RNA profiles and sub-cellular localization patterns of mRNAs in *in vitro* cellular systems (Ronander et al., 2012; Lalmansingh et al., 2013; Shaffer et al., 2013; Sinnamon and Czaplinski, 2014) and model animals such as *Drosophila* (Zimmerman et al., 2013), *Caenorhabditis elegans* (Bolková and Lanctôt, 2015), and Zebrafish (Hauptmann et al., 2016). Additionally, smRNA FISH has been used to study the subcellular localization and cell-to-cell variability of long non-coding RNAs (lncRNA); systematically quantification and categorization based on the subcellular localization patterns were achieved for a representative set of 61 lncRNAs in three different cell types (Cabili et al., 2015). Knowledge of lncRNA subcellular localization patterns is essential to understand its biological processes. An interesting application of smRNA FISH is the study on nuclear RNA foci in genetic diseases resulting from the expansion of tri-, tetra-, penta-, and hexa-nucleotide repeats; a detailed protocol was reported for detecting mRNAs containing expanded CAG and CUG repeats in fibroblasts, lymphoblasts, and induced pluripotent stem cells (Urbanek and Krzyzosiak, 2016).

Simultaneous detection of mRNA and protein quantity and their subcellular distribution in single cells by combining a RNase-free modification of the immunofluorescence (IF) technique and the smRNA FISH method observed direct interaction of RNase MCPIP1 with IL-6 mRNA (Kochan et al., 2015). Real-time live imaging using laser-scanning confocal microscope with photon-counting detectors for quantitative studies of transcription in culture cells and model animals have been achieved by smRNA-FISH and GFP-tagged reporter gene for RNA polymerase (Gregor et al., 2014). Using *Drosophila* embryo as a testing system, smRNA-FISH observed stochastic transcriptional activity of four critical patterning genes and co-packaging of transcripts as multi-copy heterogeneous granules to selected subcellular domains (Little et al., 2013, 2015). These results indicated that there are conserved mechanisms of precision mRNA transcription and localization for spatiotemporal control of protein synthesis in regulating cellular and embryo development.

## Conclusions and future directions

In summary, FISH has a wide spectrum of diagnostic and research applications as shown in Table 2. FISH has the advantage that it can be used in metaphase chromosomes and interphase nuclei, and thus offers a cell-based genetic diagnosis in complementary to DNA-based molecular testing (Xu and Li, 2013). FISH has been used as adjunctive and diagnostic assays for both constitutional and somatic cytogenomic abnormalities. FISH analysis of uncultured interphase cells from amniotic fluid or chorionic villus samples is a standard procedure for rapid prenatal testing of common aneuploidy and genomic disorders, which alleviates much anxiety for patients and physicians. The use of interphase FISH has been particularly fruitful for cancer cytogenetics, where the detection of recurrent chromosomal abnormalities and clonal evolution is crucial for classifying different types of tumors, selecting treatment protocols, and monitoring outcomes. Even with the introduction of genomic technologies like microarray analysis and exome sequencing, FISH analysis will still be an integral part of genetic diagnosis (Parisi et al., 2012; Wei et al., 2013; Martin and Warburton, 2015). Microfluidic devices for miniaturized and automatic FISH applications are currently under development (Vedarethinam et al., 2010; Kwasny et al., 2012; Kao et al., 2015). The validation of these devices in the near future and the available of more disease-specific probes will further enhance and expand the diagnostic FISH application.

**Table 2:** **FISH applications in genetic diagnosis and research**.

**Genetics diagnosis**	**References**	**Research applications**	**References**
**Constitutional chromosomal and genomic abnormalities**		**Analysis complex chromosomal rearrangements**	
Rapid screening of common aneuploidies	Ried et al., 1992	Mapping breakpoints and genomic orientation	Donnelly et al., 2010
Detection of microdeletion/microduplication syndromes	Wei et al., 2013	The study of 3D chromosomal structures	Dupont et al., 2012
Characterization of subtelomeric rearrangements	Ning et al., 1996	Define complex rearrangements	Mackinnon and Campbell, 2013
Analysis of supernumerary marker and ring chromosomes	Zhang et al., 2012	**Characterizing nuclear genomic structures**	
**Somatic recurrent chromosomal abnormalities**		Spatiotemporal organization of centromeres/telomeres	Chen et al., 2013
Detection of translocations, deletions, duplications/amplifications	Hu et al., 2014	Chromatin interaction during cell cycle	Deng et al., 2015
Monitoring disease progression and clonal evolution	Mikhail et al., 2016	*in situ chromosome haplotyping*	Beliveau et al., 2015
Assessment of sex-mismatch bone marrow transplantation	Liehr et al., 2015	**Profiling RNA transcription and localization**	
**Infectious diseases**		Quantitation of multiplex mRNAs in single cells	Lubeck et al., 2014
Detection of malaria by 16s rRNA	Shah et al., 2015	Subcellular localization of mRNAs and non-coding RNAs	Cabili et al., 2015

Novel FISH techniques and super-resolution imaging systems have been introduced to study the spatiotemporal changes of intra-nuclear genomic organization and cytoplasmic RNA profiling. These FISH techniques such as CASFISH, oligopaint-FISH, and smRNA-FISH have been developed mainly for genetic research applications. A current trend in FISH is toward simultaneous single-cell measurement of DNA, RNA, cell surface proteins, and intracellular proteins (Lai et al., 2016; Soh et al., 2016). The translation of these single molecule single cells FISH techniques into cell-based genetic diagnosis is expected to improve the analytical resolution and capacity for a spectrum of genetic defects from chromosomal and genomic abnormalities to epigenetic aberrations.

## Author contributions

CC drafted the cell-based genetics diagnosis by FISH. WS drafted the single cell DNA structural and RNA transcriptional analysis by FISH. PL organized, modified, and edited the manuscript. We would like to thank Audrey Meusel for proofreading and editing this manuscript.

### Conflict of interest statement

The authors declare that the research was conducted in the absence of any commercial or financial relationships that could be construed as a potential conflict of interest.
